# Vicarious Experience Affects Patients' Treatment Preferences for Depression

**DOI:** 10.1371/journal.pone.0031269

**Published:** 2012-02-21

**Authors:** Seth A. Berkowitz, Robert A. Bell, Richard L. Kravitz, Mitchell D. Feldman

**Affiliations:** 1 Division of General Internal Medicine, University of California San Francisco, San Francisco, California, United States of America; 2 Department of Communication, University of California Davis, Davis, California, United States of America; 3 Department of Public Health Sciences, University of California Davis, Davis, California, United States of America; 4 Division of General Medicine, University of California Davis, Sacramento, California, United States of America; King's College London, United Kingdom

## Abstract

**Purpose:**

Depression is common in primary care but often under-treated. Personal experiences with depression can affect adherence to therapy, but the effect of vicarious experience is unstudied. We sought to evaluate the association between a patient's vicarious experiences with depression (those of friends or family) and treatment preferences for depressive symptoms.

**Methods:**

We sampled 1054 English and/or Spanish speaking adult subjects from July through December 2008, randomly selected from the 2008 California Behavioral Risk Factor Survey System, regarding depressive symptoms and treatment preferences. We then constructed a unidimensional scale using item analysis that reflects attitudes about antidepressant pharmacotherapy. This became the dependent variable in linear regression analyses to examine the association between vicarious experiences and treatment preferences for depressive symptoms.

**Results:**

Our sample was 68% female, 91% white, and 13% Hispanic. Age ranged from 18–94 years. Mean PHQ-9 score was 4.3; 14.5% of respondents had a PHQ-9 score >9.0, consistent with active depressive symptoms. Analyses controlling for current depression symptoms and socio-demographic factors found that in patients both with (coefficient 1.08, p = 0.03) and without (coefficient 0.77, p = 0.03) a personal history of depression, having a vicarious experience (family and friend, respectively) with depression is associated with a more favorable attitude towards antidepressant medications.

**Conclusions:**

Patients with vicarious experiences of depression express more acceptance of pharmacotherapy. Conversely, patients lacking vicarious experiences of depression have more negative attitudes towards antidepressants. When discussing treatment with patients, clinicians should inquire about vicarious experiences of depression. This information may identify patients at greater risk for non-adherence and lead to more tailored patient-specific education about treatment.

## Introduction

Depression is common in primary care but often goes unrecognized and under-treated, with only one-fifth of patients receiving guideline concordant care [1]. Even when the diagnosis is made and treatment is initiated, as few as 25% of patients adhere to their prescribed antidepressants [1]. Low adherence to effective therapies contributes to needless patient suffering, and results in wasted time and health care expenditures.

Prior studies have examined the predictors of poor adherence [2], [3], [4], [5] and have tested various interventions aimed at improving patient adherence with antidepressant treatment [1], [6]. Notably, collaborative care models [7], [8] have demonstrated benefit by intervening on both clinician and patient level barriers to adherence. In spite of these gains, adherence to antidepressant treatment for most patients remains inadequate. Primary care physicians need better tools to identify patient attitudes and preferences for treatment that can then inform strategies for patient-specific education about antidepressants. Treatment strategies that incorporate patient preferences increase the likelihood that a patient will enter treatment, adhere to prescribed regimens, [9] and show clinical benefit [10], [11]. The determinants of patient treatment preferences for depression, however, are less well studied.

Learning theory suggests that a patient's positive past experience with depression treatment should increase motivation to seek the same or similar treatment [12]. Similarly, social cognitive theory predicts that successfully accomplishing a task should boost self-efficacy to accomplish similar tasks in the future [13], [14], [15]. Moreover, social cognitive theory posits that vicarious experience has similar effects on self-efficacy. In this context, we are considering vicarious experience to be the experience, observed by the patient, of a friend or family member with depression. Our group reported previously that physicians' vicarious experiences with depression treatment influence their attitudes and management [16], [17]. In this study, we examined whether patients' vicarious experiences with depression treatment, i.e., having a close friend or family member who had undergone treatment for depression, would lead to a more favorable attitude towards treatment.

This study was approved by the University of California, Davis, Office of Research, Institutional Review Board (IRB).

## Methods

### Ethics Statement

All co-authors of this study affirm that the research was conducted in accordance with the principles expressed in the Declaration of Helsinki. Approval to conduct this research was granted by the University of California, Davis, Office of Research, Institutional Review Board (IRB) and the University of California, San Francisco Committee on Human Research (CHR). Informed consent was obtained for all participants.

We conducted a telephone follow-up of 1054 English and Spanish speaking adult subjects from July through December 2008, randomly selected from the pool of respondents who had completed the 2008 California Behavioral Risk Factor Survey System (BRFSS). The methodology of the BRFSS has been reported elsewhere [18]. Because the focus of the current survey was on attitudes toward and experience with depression, subjects with a history of depression were over-sampled (approximately threefold) to yield an adequate sample size for those with a depression history. Respondents were asked a set of questions about their depression-related beliefs. From these responses we constructed an outcome measure of attitude towards antidepressant therapy based on 6 items (Cronbach's alpha = .78) (Appendix S1: Item Analysis for Outcome Variable). These items were selected based on a factorial analysis demonstrating unidimensionality, with all items having factor loadings ≥.65. This outcome variable had possible scores from 6 to 30, with a higher value representing a more positive attitude towards antidepressants.

Respondents were also asked if they had ever been treated for depression and completed the PHQ-9, a measure of current depressive symptoms [19]. Those individuals who were undergoing treatment at the time of the survey or had ever been treated for depression in the past were asked to rate the success of their treatment on a 3-point scale (1 = not very successful, 2 = somewhat successful, 3 = very successful). Vicarious experiences with depression were assessed with two questions about whether the respondent knew of a friend or family member who had been treated for depression. Responses to these items were coded dichotomously (no or yes).

Standard demographic variables were assessed to characterize the sample and as statistical controls. These included gender, age, race (nonwhite/white), Hispanic cultural identification, education, income, and relationship status. Healthcare status was addressed by asking if the respondent had health insurance and a regular source of primary care. Descriptive statistics were used to characterize the sample. Because presumably patients who have a personal history of depression think about treatment from a fundamentally different perspective than those who do not, separate linear regression analyses were carried out for individuals with versus without a history of depression to evaluate the relationship of having a friend or family member with a depression history on attitudes toward antidepressant treatment. Analyses were performed using STATA 11.1 (College Station, TX).

## Results

Table 1 reports sample demographic and health characteristics. Our sample was 68% female, 91% white, and 13% Hispanic (patients could self-identify as both white and Hispanic). Respondents ranged in age from 18–94 (mean: 56.4 years). A majority were married or were in a committed relationship (55%); 52% had graduated from college, and 42% had an annual household income under $50,000/year. Almost half of respondents (45%) had been treated for depression and 21% were undergoing treatment at the time of the survey. Many respondents reported knowing a friend (64%) or family member (53%) who had undergone treatment for depression. Most had health insurance (93%) and a regular source of health care (88%). The mean PHQ-9 score was 4.33. Approximately 15% of respondents had a PHQ-9 score >9.0 at the time of the survey, consistent with active depressive symptoms [19].

**Table 1:** Demographics and Health Characteristics.

Respondent Characteristic	%	N
DEMOGRAPHIC MEASURES		
Female	67.7	714
White Race	90.7	954
Hispanic Identification	12.7	134
Age		
18–29	4.5	47
30–39	9.2	97
40–49	19.6	207
50–59	22.9	241
≥60	43.8	461
Married or Partnered	54.8	578
Education		
H.S. or Less	18.1	191
Some College/Technical School	29.6	312
College Graduate	52.2	550
Household Income		
Under $20,000	15.6	164
$20,000–$34,999	13.9	146
$35,000–$49,999	12.3	130
$50,000–$74,999	16.2	171
$75,000–$100,000	17.3	182
>$100,000	22.1	233
Unsure/Declined To Answer	2.7	28
DEPRESSION-RELATED MEASURES		
Ever Treated for Depression	45.1	475
Currently Under Treatment for Depression	21.6	228
Has Friend Treated for Depression	64.3	670
Has Family Member Treated For Depression	52.7	555
Has Health Insurance	93.7	988
Regular Source of Primary Care	78.7	830
PHQ-9 Score >9	14.5	153

Separate analyses were carried out for respondents with versus without a personal history of depression. In both analyses, we controlled for current depression symptoms and socio-demographic factors. In respondents both with (coefficient 0.90, p = 0.15) and without (coefficient 0.96, p = 0.005) a personal history of depression, having a vicarious experience with depression, that is, knowing a friend or family member treated for depression, was associated with a more favorable attitude towards antidepressant medications.

To further delineate the relationship between vicarious experience and treatment preferences, we then performed a more detailed regression to examine separately the influence of friend and family experience. Results are presented in table 2. For respondents with a personal history of depression, having a *family member* who had been treated for depression was associated with having a positive attitude toward antidepressant medications (coefficient 1.08, p = 0.03). However, among respondents with no personal history of depression, having a *friend* who had been treated for depression was associated with having a positive attitude toward antidepressant medication (coefficient 0.77, p = 0.03). Additionally, a history of prior treatment with antidepressant medication is strongly associated with having a more favorable attitude to antidepressants. Interestingly, a higher PHQ-9 score is *negatively* associated with attitudes towards anti-depressants, suggesting that those who are more depressed are less favorably inclined towards medication.

**Table 2:** Results of linear regression analyses predicting attitude toward antidepressants with subgroups of vicarious experience of depression for respondents with and without a history of depression.

Covariate	Coeff.	95% CI	P
HISTORY OF DEPRESSION (n = 451)			
Has Friend With History of Depression	0.16	(−0.94, 1.27)	0.77
Has Family Member With History of Depression	1.08	(0.08, 2.07)	0.03
Past history of medication use	3.00	(1.98, 4.03)	<0.0001
PHQ-9 Score	−0.07	(−0.14, −0.001)	0.045
NO HISTORY OF DEPRESSION (n = 550)			
Has Friend With History of Depression	0.77	(0.06, 1.48)	0.03
Has Family Member With History of Depression	0.47	(−0.25, 1.21)	0.20
PHQ-9 Score	−0.20	(−0.31, −0.10)	<0.0001

Note: Results have been adjusted for the following control variables: Age, PHQ-9 score, income, educational attainment, gender, ethnicity, availability of primary care, and relationship status.

A positive coefficient represents a more positive attitude towards antidepressant medications.

Number of observations in each regression model differs slightly from demographic data due to missing responses.

## Discussion

This investigation suggests that having a vicarious experience with depression may lead to a more positive attitude towards treatment with antidepressant medications. Specifically, having a family member who had been treated for depression was associated with positive attitudes toward antidepressants for respondents with a history of depression. In contrast, for respondents with no history of depression treatment, having a friend with a history of depression was associated with positive attitudes toward antidepressant medications. These findings support our hypothesis, based on learning and social cognitive theory, that both personal past experiences and the experiences of others can significantly affected attitudes towards treatment.

Patients who have taken antidepressant medication in the past have a highly favorable attitude towards antidepressants. This is consistent with other work which has shown an favorable attitude towards treatment for those who have been prescribed or are currently taking anti-depressants [20],. Prior research suggests that those with positive attitudes towards antidepressants are more likely to be adherent [21] and that as treatments more closely match patient preferences, adherence is increased [10].

Interestingly, we also found that higher scores on the PHQ-9 are associated with more negative attitudes. As past work has shown that those with negative attitudes towards treatment have decreased adherence [22], this suggests that those who might benefit most from treatment, those most depressed [23], might be a group at particular risk for non-adherence. Every effort should be made to engage these patients in evidence based therapies.

Busy clinicians, especially in primary care, often face multiple demands for their time. We feel the ideal time to discuss vicarious experiences with depression treatment with a patient is when a clinician is considering initiation of treatment. This can be incorporated into routine counseling and anticipatory guidance around starting a new medication. Open-ended statements encourage patients to share details about their own experiences. For example, clinicians could say “Do you know anyone who has had depression?” or “Tell me about people you have known with depression”. Once obtained, this information can help identify patients at greater risk for non-adherence and be used to tailor patient-specific education about treatment.

It is curious that patients with a personal history of depression are influenced differently by their vicarious experience compared to those with no personal history. Patients with a personal and family history of depression may view their condition as more attributable to heredity and thus be open to a biomedical perspective, one that views pharmacological treatment favorably. In contrast, a person with no history of depression may see the depression experiences of a friend as being more informative than the experience of a family member. It should be noted, however, that although the association between attitudes toward antidepressant medications and having a family member who had been treated for depression for this group of respondents missed statistical significance, the relationship was in the anticipated direction. How vicarious experience mediates attitudes toward treatment for depressive symptoms is unknown. One possible mechanism may lie through the reduction of stigma. Prior studies of vicarious experience have noted that people who have a friend or family member with depression rate lower on measures of stigma (such as regarding depression as a ‘real’ illness, or feeling that a person with depression could “get better if they wanted”) than those without such an experience [24].

This study has several limitations. First, our outcome was a psychometric measure of attitudes towards antidepressants derived from survey data. It was not validated or assessed for reliability in other populations, and was not a clinical measure. Thus we cannot be sure how the associated positive regard will translate in the office setting. Second, our data are cross-sectional and thus can demonstrate an association between vicarious experience and treatment attitudes, but not a causal link. Third, compared to the BRFSS survey sample as a whole [25], which is representative of California, our sample was generally older and had higher income than the general population. Although we controlled for age and income in our analysis, this may limit generalizability to other settings.

In summary, patients who lack personal or vicarious experiences with depression tend to have negative attitudes towards antidepressants. Conversely, having such experience may facilitate acceptance of pharmacotherapy. Future research should focus on strategies that utilize knowledge of patient characteristics to boost treatment adherence.

## Supporting Information

Appendix S1 **Item Analysis for Outcome Variable.**
(DOC)
