# Supplementary material for: Vicarious Experience Affects Patients' Treatment Preferences for Depression
Source: PLoS One. 2012 Feb 21;7(2):e31269. doi: 10.1371/journal.pone.0031269 (PMC3283627; doi:10.1371/journal.pone.0031269)
Supplement: Appendix S1 — Item Analysis for Outcome Variable. (DOC) [file pone.0031269.s001.doc]

Appendix S1: Item Analysis for Outcome Variable

| **Item-Total Statistics** | | | | |
| --- | --- | --- | --- | --- |
|  | **Scale Mean if Item Deleted** | **Scale Variance if Item Deleted** | **Corrected Item-Total Correlation** | **Cronbach's Alpha if Item Deleted** |
| ANTIDEPRESSANT MEDICATIONS PREVENT PERSON FROM EXPERIENCING NATURAL SADNESS | 16.78 | 13.447 | .547 | .744 |
| ANTIDEPRESSANT MEDICATIONS CAN MAKE PEOPLE FEEL NUMB | 17.03 | 13.479 | .532 | .747 |
| ANTIDEPRESSANT MEDICATIONS ARE ADDICTIVE | 17.00 | 13.530 | .533 | .747 |
| IT WOULD VERY INCONVENIENT TO TAKE MEDS EVERYDAY FOR DEPRESSION | 16.71 | 12.911 | .488 | .763 |
| MEDICATION IS HIGHLY EFFECTIVE IN TREATING DEPRESSION | 16.31 | 14.271 | .502 | .755 |
| TAKING ANTI-DEPRESSANTS ON A DAILY BASIS IS GENERALLY SAFE | 16.62 | 13.624 | .594 | .734 |
